# Primary Debulking Surgery Versus Neoadjuvant Chemotherapy in Advanced Ovarian Cancer: A Meta-Analysis of All Randomized Clinical Trials with Subgroup Analysis by Patient Profile

**DOI:** 10.32604/or.2026.074934

**Published:** 2026-04-22

**Authors:** Carlo Ronsini, Giuseppe Cucinella, Maria Cristina Solazzo, Mariano Catello Di Donna, Cono Scaffa, Stefano Cianci, Manuela Ludovisi, Sandro Pignata, Vito Chiantera

**Affiliations:** 1 Unit of Gynecologic Oncology, National Cancer Institute, IRCCS, Fondazione “G. Pascale”, Naples, Italy; 2 Unit of Gynecology and Obstetrics, Policlinico “G. Martino”, Department of Human Pathology of Adult and Childhood “G. Barresi”, University of Messina, Messina, Italy; 3 Department of Life, Health and Environmental Sciences, University of L’ Aquila, L’ Aquila, Italy; 4 Medical Oncology Division B, National Cancer Institute, G Pascale Foundation, Naples, Italy

**Keywords:** Advanced ovarian cancer, primary cyoreductive surgery, neoadjuvant chemotherapy, cytoreductive surgery, randomized controlled trials

## Abstract

**Background:**

The optimal sequencing of surgery and chemotherapy in advanced epithelial ovarian cancer remains debated. While primary debulking surgery (PDS) has been considered the standard approach, recent randomized trials have questioned its survival advantage over neoadjuvant chemotherapy (NACT) followed by interval debulking surgery (IDS). The study aimed to systematically evaluate phase III randomized controlled trials comparing PDS and NACT.

**Methods:**

Following PRISMA guidelines (PROSPERO ID 1169057), PubMed and Scopus were systematically searched in October 2025 for phase III randomized clinical trials evaluating cytoreductive strategies in ovarian carcinoma. Only full-text English studies reporting overall survival (OS) or disease-free survival (DFS) were included. Risk ratios (RR) with 95% confidence intervals (CI) were calculated.

**Results:**

Five phase III trials (EORTC 55971, CHORUS, JCOG0602, SCORPION, TRUST) comprising 2296 patients met the inclusion criteria. PDS (n = 1139) and NACT (n = 1157) showed comparable OS (RR 0.99, 95% CI 0.94–1.03, *p* = 0.55, *I*^2^ = 0%) and DFS (PDS RR 0.98, 95% CI 0.95–1.02, *p* = 0.27, *I*^2^ = 0%). Subgroup analyses confirmed the absence of significant differences for patients with CC0 (RR 0.96, 95% CI 0.87–1.05, *p* = 0.35, *I*^2^ = 0%), FIGO stage III disease (RR 0.97, 95% CI 0.92–1.03, *p* = 0.34, *I*^2^ = 0%), or age under 70 years (RR 1.03, 95% CI 0.97–1.09, *p* = 0.38, *I*^2^ = 0%).

**Conclusions:**

PDS and NACT provide no significant survival outcomes in advanced ovarian cancer. No clear survival benefit for PDS was observed. Refinement of patient selection, integration of predictive biomarkers, and re-evaluation of PDS in the context of HIPEC and Poly-ADP-Ribose Polymerase (PARP) inhibitor use are warranted to guide individualized treatment strategies.

## 1 Introduction

Epithelial ovarian, tubal, and primary peritoneal cancers represent the most lethal gynecologic malignancies, with the majority of patients diagnosed at an advanced stage (FIGO IIIC–IV) [1,2]. Historically, primary debulking surgery (PDS) followed by adjuvant platinum-taxane chemotherapy has been regarded as the cornerstone of management, based on the well-established correlation between the extent of residual disease and survival outcomes [3,4]. However, complete cytoreduction is not always feasible, particularly in patients with extensive upper abdominal disease or poor performance status [5]. To improve operability and reduce perioperative morbidity, an alternative strategy, neoadjuvant chemotherapy (NACT) followed by interval debulking surgery (IDS), was developed [6,7]. The pivotal EORTC 55971 trial [8] and the CHORUS trial [9] were the first large, multicenter randomized studies to demonstrate that NACT followed by IDS was non-inferior to PDS in terms of overall and progression-free survival (PFS), with fewer postoperative complications and lower mortality rates. These trials, however, mainly enrolled unselected advanced-stage populations, without preoperative assessment of resectability, and included a substantial proportion of patients with suboptimal cytoreduction rates in the PDS arm, potentially limiting the generalizability of their conclusions to specialized surgical centers. Subsequent studies sought to address these limitations. The JCOG 0602 trial [10] conducted in Japan confirmed similar survival outcomes between PDS and NACT. However, it failed to demonstrate the non-inferiority of NACT, suggesting that PDS may still offer benefit when optimal cytoreduction is achievable. Likewise, the SCORPION trial [11] adopted a rigorous preoperative selection through laparoscopic assessment of tumor load. It showed comparable survival between arms and highlighted that, when performed at maximal surgical effort, both strategies yield no significant different efficacy, albeit with different toxicity profiles. Most recently, the TRUST trial [12] has provided contemporary evidence from high-volume centers, confirming the absence of a significant survival advantage of PDS over NACT in the overall population. Nevertheless, subgroup analyses from TRUST suggested a potential trend favoring PDS in well-selected patients, those with limited intra-abdominal disease and a high likelihood of complete cytoreduction, thereby reigniting the debate about patient selection and surgical radicality in the era of personalized oncologic surgery. Recent therapeutic advances, such as the incorporation of HIPEC [13] at interval debulking surgery and the use of PARP inhibitors as maintenance therapy [14], are progressively reshaping outcomes in advanced ovarian cancer. Although these approaches were not included in the historical randomized trials comparing PDS and NACT, they highlight the need to interpret surgical strategies within the framework of an evolving multimodal treatment paradigm.

***Objectives***

The objective of this study is to systematically analyze all phase III randomized clinical trials comparing PDS and NACT in patients with advanced epithelial ovarian, tubal, or peritoneal cancer. Beyond synthesizing survival and morbidity outcomes, we aim to perform subgroup analyses focusing on highly selected patient populations, specifically, women with disease confined to the abdomen, those who achieved complete macroscopic cytoreduction (CC0), and those younger than 70 years. This approach seeks to clarify whether PDS may still provide oncologic advantages in patients most likely to benefit from an aggressive surgical approach under optimal selection criteria.

## 2 Materials and Methods

The methods for this study were specified a priori based on the recommendations in the Preferred Reporting Items for Systematic Reviews and Meta-Analyses (PRISMA) statement (Supplementary Material) [15]. We registered the Review to the PROSPERO site for meta-analysis with protocol number ID 1169057. The study protocol was registered in PROSPERO prior to data extraction and statistical analysis.

The research question was structured according to the PICOS framework:

**Population (P):** women with FIGO stage III–IV epithelial ovarian, tubal, or peritoneal cancer;

**Intervention (I):** PDS followed by platinum–taxane chemotherapy;

**Comparison (C):** NACT followed by IDS;

**Outcomes (O):** overall survival (OS) and disease-free survival (DFS);

**Study Design (S):** Randomized Clinical Trials.

### 2.1 Search Method

A systematic literature search was conducted to identify randomized clinical trials evaluating cytoreductive surgery in patients with ovarian, tubal, or peritoneal cancer. The search was performed in the PubMed and Scopus databases in October 2025. No restrictions were applied regarding publication date or study location. Only full-text articles published in English were included. The search strategy employed the MeSH term “Ovarian Neoplasms” in both databases. Using only one search term allowed us to keep the search field as broad as possible.

### 2.2 Study Selection

Study selection was performed independently by two reviewers (Stefano Cianci and Manuela Ludovisi). In cases of disagreement, the final decision regarding inclusion or exclusion was made by Carlo Ronsini.

The inclusion criteria were as follows: (1) studies enrolling patients with a primary diagnosis of ovarian, tubal, or peritoneal cancer who underwent surgical treatment; (2) studies reporting at least one relevant outcome, including OS or DFS; (3) original peer-reviewed publications; and (4) phase III randomized clinical trials.

We excluded non-original works, preclinical or animal studies, conference abstracts without full-text publication, and articles not written in English. When feasible, corresponding authors of studies available only as congress abstracts were contacted via email to request additional data. This was only necessary in the case of the TRUST trial, that was included with the original data.

**Figure 1:**
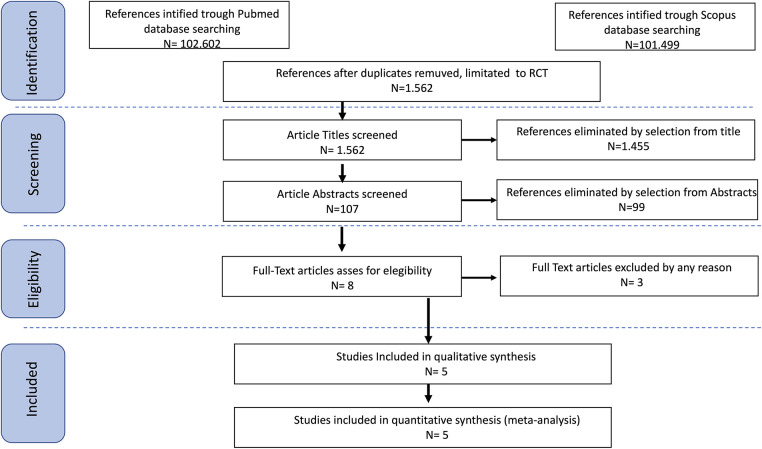
PRISMA flow chart.

All included and excluded studies, along with reasons for exclusion, were detailed in the PRISMA flow diagram (Fig. 1). Potential conflicts of interest were assessed for each included study.

### 2.3 Data Extraction

Data extraction was independently performed by Giuseppe Cucinella and Mariano Catello Di Donna using a standardized template. Extracted information included study characteristics, population details, intervention and control definitions, survival outcomes, follow-up duration, and reported surgical morbidity. Any discrepancies were reviewed by Carlo Ronsini for accuracy.

### 2.4 Quality Assessment

The methodological quality of the included studies was evaluated using the Newcastle–Ottawa Scale (NOS) [16]. This tool assesses studies based on three main domains: selection, comparability, and exposure, with total scores ranging from 0 (indicating the lowest quality) to 9 (indicating the highest quality). Two reviewers (Carlo Ronsini and Stefano Cianci) independently appraised the quality of each study, and any discrepancies were resolved through discussion or, when necessary, by consulting a third reviewer (Vito Chiantera). Although the Newcastle–Ottawa Scale (NOS) was originally developed for observational studies, it was used in this review as a structured tool to provide a global appraisal of study quality and reporting consistency. Assessment of internal validity and risk of bias specific to randomized trials was performed separately using the Cochrane RoB 2 tool. Details of the NOS assessments are provided in the Table A1.

### 2.5 Risk of Bis Assessment

The methodological quality of the included trials was assessed using the Cochrane Risk of Bias 2 (RoB 2) tool, with evaluation conducted at the domain level (randomization process, allocation concealment, blinding, completeness of outcome data, and selective reporting), and an overall risk-of-bias judgment subsequently assigned for each study in accordance with Cochrane guidance. The assessment was performed independently by two authors (Mariano Catello Di Donna and Maria Cristina Sollazzo), and any disagreements were resolved by consensus. Key trial design characteristics, including primary endpoints, statistical framework, power assumptions, and surgical quality indicators, are summarized in Table A2.

### 2.6 Statistical Analysis

Heterogeneity across studies was assessed using the Chi-square and *I*^*2*^ statistics. Risk ratios (RR) with 95% confidence intervals (CI) were calculated for dichotomous outcomes. A fixed-effect model was applied when heterogeneity was not significant (*I*^2^ < 50%), while a random-effects model was used otherwise (*I*^2^ ≥ 50%). The main clinical endpoints were OS and DFS. DFS was defined as the interval between surgery and disease recurrence or the last available follow-up, while OS was defined as the time from surgery to death from disease or last follow-up. Chi-square tests were used to compare categorical variables. Subgroup analyses were performed for patients with FIGO stage III disease, those aged under 70 years, and those with no residual macroscopic disease after surgery (CC0). Although HR are generally preferred for time-to-event outcomes, their use was not feasible in this meta-analysis due to incomplete reporting of survival statistics across trials. Consequently, RR was adopted as a pragmatic approach to maximize data availability, acknowledging the inherent limitations of this choice. A sensitivity analysis excluding the TRUST trial was not performed, as the number of available randomized phase III trials was limited and exclusion of the largest and most contemporary study would have substantially reduced statistical power and precision of the pooled estimates, particularly for subgroup analyses. All statistical analyses were conducted using R software (version 2024.12.0 + 467) and RStudio (version 2024.12.0 + 467). The R packages meta and metafor were employed for meta-analytical procedures. A *p*-value < 0.05 was considered statistically significant.

## 3 Results

### 3.1 Studies’ Characteristics

After the database search, 1562 articles matched the search criteria. After manually removing records with no full text, duplicates, and wrong study designs (other than randomized clinical trials), 8 were suitable for eligibility. Of those, 5 matched the inclusion criteria and were included in the meta-analysis. The countries where the studies were conducted, the publication year range, the FIGO stages of ovarian, tubal, or primary peritoneal cancer, and the number of participants are summarized in Table 1.

**Table 1:** Studies included.

Randomized Clinical Trials							
Registration Number	Year of Publication	Identification Name	Country	Study Year	FIGO Stage	N° of Partecipant	Mean FUP* Months
NCT00003636	2010	EORTC [8]	Europe	1998–2008	IIIC–IV	632	56, 4
ISRCTN74802813	2015	CHORUS [9]	UK-New Zeland	2004–2010	III–IV	550	52, 8
UMIN000000523	2020	JCOG0602 [10]	Japan	2006–2011	III–IV	301	58
NCT01461850	2020	SCORPION [11]	Italy	2011–2016	III–IV	171	59
NCT02828618	Under Publication	TRUST [12]	Europe	2019–2024	IIIB–IV	688	56

Note: °Number, *Follow Up, FIGO: International Federation of Gynecology and Obstetrics.

The quality of all studies was assessed by NOS (Table A1). Overall, the inclusion years ranged from 1998 to 2024. Follow up period ranged from 52 to 59 months on average.

### 3.2 Treatment Comparison

Across all included randomized phase III trials, two main therapeutic strategies were compared for patients with advanced-stage (FIGO III–IV) ovarian, tubal, or primary peritoneal carcinoma.

The first approach consisted of PDS followed by adjuvant platinum–taxane chemotherapy, generally six to eight cycles of carboplatin (AUC 5–6) plus paclitaxel (175 mg/m^2^ every three weeks). The second strategy involved NACT, understood as 3 to 4 cycles of the same platinum-taxane regimen, followed by IDS in patients with stable or responding disease, and then completion of chemotherapy for a total of 6 to 8 cycles.

All protocols aimed for complete cytoreduction (no macroscopic residual disease). Surgical procedures included hysterectomy, bilateral salpingo-oophorectomy, omentectomy, and resection of visible metastases, performed by specialized gynecologic oncologists.

While the chemotherapy regimen and surgical intent were similar across studies, designs differed in patient selection: EORTC [8] and CHORUS [9] enrolled unselected advanced cases, without preoperative laparoscopic assessment of resectability, JCOG 0602 [10] limited inclusion to imaging-confirmed stage III–IV disease without diagnostic laparoscopy, and SCORPION [11] and TRUST [12] specifically randomized patients with high tumor load confirmed by laparoscopic assessment.

### 3.3 Meta-Analysis

**Figure 2:**
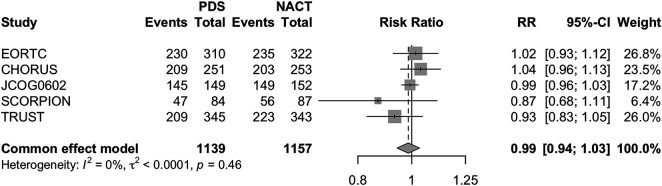
Forest Plot of Overall Survival (OS) for primary debulking surgery (PDS) and NACHT. Forest plot of OS comparing PDS vs. neoadjuvant chemotherapy followed by interval debulking surgery (NACT–IDS). Risk ratios (RRs) with 95% confidence intervals (CIs) are reported. A fixed-effects model was used due to negligible heterogeneity (*I*^2^ = 0%).

A total of 2296 patients were analyzed. 1139 patients in the Primary Debulking Group arm were compared with 1157 patients who underwent Neoadjuvant chemotherapy followed by interval debulking surgery, exploring OS and DFS outcome. Because of low heterogeneity (*I*^2^ = 0%; *t* = 0.0014), the fixed-effects model was applied. The OS was almost identical in the two groups, without reaching statistical significance (PDS RR 0.99 [95% CI 0.94–1.03] *p* = 0.55) (Fig. 2).

**Figure 3:**
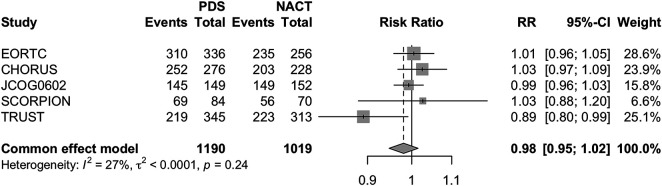
Forest Plot of Disease Free Survival (DFS) for PDS and NACHT Forest plot of DFS comparing PDS vs. neoadjuvant chemotherapy followed by NACT–IDS. RRs with 95% CIs are reported. A fixed-effects model was used due to negligible heterogeneity (*I*^2^ = 0%).

The same analysis conducted for the DFS did not reveal a statistically significant difference between the two approaches. (PDS RR 0.98 [95% CI 0.95–1.02] *p* = 0.27) (Fig. 3).

**Figure 4:**
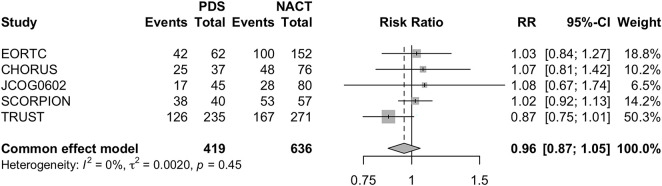
Forest Plot of OS in patients with CC0 for PDS and NACHT Forest plot of OS in patients with complete cytoreduction (CC0) comparing PDS vs. neoadjuvant chemotherapy followed by NACT–IDS. RRs with 95% CIs are reported. A fixed-effects model was used due to negligible heterogeneity (*I*^2^ = 0%).

To investigate the effects of the two different therapeutic strategies in selected settings of hypothetically more suitable patients for surgery, we conducted sub-analyses. In particular, a first sub-analysis was conducted on the OS of patients without macroscopic post-surgical tumor residue (CC0). Even in this patient setting, PDS failed to show clear advantages over NACT (PDS RR 0.96 [95% CI 0.87–1.05] *p* = 0.35) (Fig. 4).

**Figure 5:**
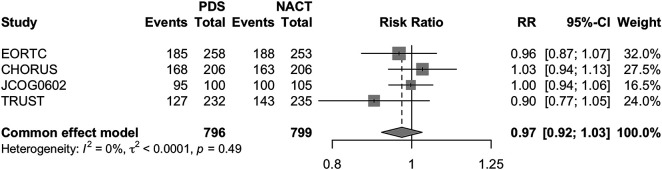
Forest Plot of OS in patients with International Federation of Gynecology and Obstetrics (FIGO) Stage III for PDS and NACHT. Forest plot of overall survival in patients with Stage III cancer comparing PDS vs. neoadjuvant chemotherapy followed by NACT–IDS. RRs with 95% CIs are reported. A fixed-effects model was used due to negligible heterogeneity (*I*^2^ = 0%).

A second sub-analysis was conducted on the OS of patients with FIGO stage limited to III. In this analysis data from Scorpion were not available. Even in this group of patients, the PDS group did not show a clear advantage over the NACT group. (PDS RR 0.97 [95% CI 0.92–1.03] *p* = 0.34) (Fig. 5).

**Figure 6:**
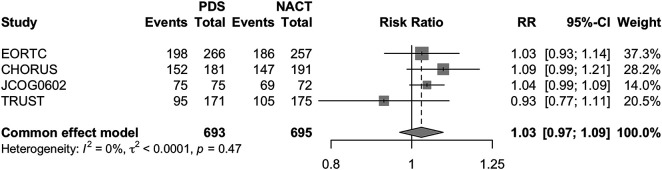
Forest Plot of OS in patients under 70 years old for PDS and NACHT Forest plot of overall survival in patients younger than 70 years old comparing primary debulking surgery (PDS) vs. neoadjuvant chemotherapy followed by interval debulking surgery (NACT–IDS). Risk ratios (RRs) with 95% confidence intervals (CIs) are reported. A fixed-effects model was used due to negligible heterogeneity (*I*^2^ = 0%).

Finally, a last sub-analysis was limited to patients under the age of 70, with similar results (PDS RR 1.03 [95% CI 0.97–1.09] *p* = 0.38) (Fig. 6).

Funnel plot analysis did not reveal significant publication bias. Sensitivity analyses excluding each trial sequentially confirmed the robustness of the pooled estimates. However, confidence intervals remained compatible with clinically relevant differences in either direction.

## 4 Discussion

### 4.1 Interpretation of Results

Across all phase III randomized trials comparing PDS and NACT, survival outcomes appeared broadly similar across treatment strategies; however, the available evidence does not allow definitive conclusions regarding true equivalence. Both treatment strategies achieved similar progression-free and OS when maximal cytoreduction was pursued, confirming that the extent of residual disease remains the strongest prognostic factor, irrespective of treatment sequence; nevertheless, the limitations of aggregated trial-level data preclude any formal claim of equivalence. When focusing on highly selected subgroups, such as patients with disease confined to the abdomen, those younger than 70 years, or those who achieved complete cytoreduction, no statistically significant survival advantage emerged for PDS, although a slight trend toward improved outcomes was observed. This suggests that even in favorable clinical settings, the oncologic superiority of PDS remains uncertain, and its benefit may be limited to a narrow subset of optimally selected patients. Although a slight trend favoring PDS was observed in some subgroups, this signal should be interpreted with caution. The reliance on aggregated rather than individual patient data limits adjustment for important prognostic factors, and minor imbalances across trials may produce apparent differences that are not clinically meaningful. Conversely, NACT continues to represent a valid and often safer alternative, particularly in cases with high tumor load, extra-abdominal involvement, or reduced performance status [11,17]. These findings underscore that the choice between PDS and NACT should be guided by individual patient factors, surgical feasibility, and institutional expertise, rather than by a presumed universal advantage of one approach over the other. Importantly, the absence of statistically significant differences should not be interpreted as proof of equivalence between PDS and NACT, but rather as an indication of insufficient evidence to demonstrate the superiority of one strategy over the other within the constraints of the available data. It should be emphasized that the pooled estimates derive from trials with heterogeneous designs, including differences in primary endpoints (OS vs. PFS), statistical frameworks (non-inferiority vs. superiority), and patient selection criteria. Moreover, some trials were underpowered to detect modest but clinically meaningful differences. As a consequence, the observed confidence intervals remain compatible with potential benefit in either direction, and formal equivalence between PDS and NACT cannot be established.

### 4.2 Clinical Implication

From a clinical standpoint, these findings support a personalized approach in the management of advanced ovarian, tubal, and peritoneal cancer. The absence of a clear survival advantage for PDS, even among well-selected patients, highlights that the decision between upfront surgery and neoadjuvant chemotherapy should be individualized, taking into account tumor burden, disease distribution, patient performance status, and surgical expertise. While PDS may still be considered in patients with a high likelihood of complete cytoreduction, its benefits appear modest and must be balanced against the higher surgical morbidity associated with extensive cytoreductive procedures [18]. Conversely, for sure, NACT provides a safer and equally effective option in patients with bulky disease or limited operability, without compromising OS [11]. It has recently been highlighted that continuing treatment beyond four cycles may also be a useful strategy if pursued to achieve complete cytoreduction, which remains the main prognostic factor in this disease [19]. Importantly, current subgroup analyses have not yet succeeded in identifying the truly “ideal” candidate for PDS: a younger patient with disease confined to the abdomen and amenable to complete resection. In such a profile, the theoretical advantage of upfront surgery remains biologically plausible but unproven, representing an area of clinical and translational interest for future prospective research. Collectively, these data emphasize that treatment strategy should prioritize achieving complete cytoreduction by the safest and most effective route [20], rather than rigidly adhering to a surgical-first paradigm. Accordingly, this meta-analysis cannot establish equivalence between PDS and NACT, but rather highlights the lack of conclusive evidence demonstrating the oncologic superiority of either approach in the context of contemporary patient selection and treatment strategies.

### 4.3 Comparison with Existing Literature

Our findings are consistent with the major randomized clinical trials in this field. However, it is important to emphasize that primary cytoreductive surgery followed by adjuvant chemotherapy remains the worldwide standard of care, as decades of observational and multi-institutional evidence consistently demonstrate that complete cytoreduction at upfront surgery is associated with the best long-term survival outcomes, and the methodological limitations of the available randomized trials should therefore be taken into account when interpreting their results. Moreover, the intrinsic nature of these studies, conducted over long timeframes and within evolving medical contexts, introduces several gray areas that limit their direct comparability. In 2018, van Driel et al. demonstrated that the addition of hyperthermic intraperitoneal chemotherapy (HIPEC) at the time of interval debulking surgery significantly improved both DFS andOS [13,21–23]. This therapeutic option was not included in any of the trials analyzed in the present review, creating a mismatch with the current standards of care for epithelial ovarian cancer [24]. Similarly, the introduction of PARP inhibitors as maintenance therapy in patients with homologous recombination deficiency (HRD) has profoundly changed the prognosis of advanced disease, leading to substantial improvements in both OS and DFS [25–27]. The absence of standardized maintenance therapy across the historical randomized trials represents a major limitation to the clinical applicability of their results in today’s therapeutic landscape. Notably, the increasing use of HIPEC at interval debulking surgery and the widespread adoption of PARP inhibitors as maintenance therapy are progressively reshaping the therapeutic landscape of advanced ovarian cancer. These modalities, which were not incorporated into the historical randomized trials included in this analysis, may substantially influence long-term outcomes and should be considered when interpreting the applicability of past evidence to contemporary practice. Another critical issue common to all studies is the high proportion of patients who did not achieve complete cytoreduction (CC0), which remains the most crucial determinant of long-term survival [3,20,27]. Although our subgroup analysis did not show a statistically significant survival benefit for PDS, it revealed a trend that could become clinically relevant with larger sample sizes. This finding underlines the importance of optimizing preoperative assessment of resectability to improve surgical outcomes. Significant progress in this area has been made by the Italian group led by Fagotti, who pioneered the routine use of laparoscopic evaluation to predict the likelihood of complete cytoreduction [28–30]. Nevertheless, even this strategy does not guarantee 100 percent CC0 resections, emphasizing that any therapeutic approach must demonstrate reproducibility in real-world settings. Ultimately, treatment choice should be guided by the goal that is most realistically achievable for each individual patient, rather than by theoretical expectations of maximal cytoreduction. The integration of molecular biomarkers, such as BRCA and HRD status, and radiologic tools like laparoscopy-based predictive models, may help refine patient selection for optimal treatment sequencing in the near future.

### 4.4 Strengths and Limitations

The main strength of this analysis lies in the comprehensive inclusion of all available phase III randomized trials comparing PDS and NACT. By integrating evidence from studies conducted in different populations and clinical settings, this work provides a balanced and updated overview of the long-standing debate on the optimal sequencing of treatment in advanced epithelial ovarian, tubal, and peritoneal cancer. Another important aspect is the attempt to explore specific subgroups of patients, focusing on clinical factors that may influence the oncologic impact of PDS, such as age, disease distribution, and completeness of cytoreduction. However, several limitations should be acknowledged. First, there is substantial heterogeneity among the included trials in terms of surgical expertise, patient selection, and criteria used to define optimal cytoreduction. Second, the subgroup analyses were based on aggregated data rather than individual patient data, which limits the ability to identify minor but potentially relevant differences. A major limitation of the present meta-analysis is the reliance on aggregated published data rather than individual patient data (IPD). Without IPD, it is not possible to uniformly adjust for key prognostic factors, explore detailed interactions between baseline characteristics and treatment effect, or account for center-level variability. This constraint may reduce the granularity and precision of subgroup analyses and limit the ability to draw definitive conclusions regarding which patients may truly benefit from PDS vs. NACT. Third, changes in clinical practice over time, including improvements in perioperative care, advances in imaging, and the introduction of HIPEC and PARP inhibitors, have significantly modified the current therapeutic scenario, reducing the direct applicability of older studies. Another important consideration is the potential for temporal bias. The randomized trials included in this meta-analysis span more than two decades, during which significant advances in surgical expertise, anesthetic techniques, perioperative care, and institutional infrastructure have occurred. These improvements may have influenced surgical outcomes and complication rates over time, limiting the comparability of older studies with more contemporary practice. A further limitation of this meta-analysis is the impossibility of assessing absolute 10-year survival, which could serve as an important indicator of cure in advanced ovarian cancer. Updated long-term follow-up data are not yet available for several of the most recent phase III trials, and many patient cohorts have not reached the 10-year timepoint. Additionally, the absence of individual patient data prevents stratification of long-term outcomes by residual disease status. As a result, 10-year survival could not be evaluated in a standardized and comparable way across studies. An additional limitation relates to the inclusion of the TRUST trial, which at the time of analysis was not yet fully published. Although its inclusion provides valuable contemporary data from high-volume centers, conclusions incorporating TRUST results should be regarded as provisional until full peer-reviewed publication becomes available. Nevertheless, TRUST findings were directionally consistent with previously published randomized trials, suggesting that its inclusion is unlikely to qualitatively alter the overall interpretation of the evidence. Despite these limitations, the consistency of findings across trials supports the robustness of the conclusions. Taken together, the available evidence indicates that there is no conclusive proof of oncologic superiority for PDS over NACT, even in selected patients.

## 5 Conclusion

Overall, evidence from phase III randomized trials shows no significant survival advantage of PDS over NACT in advanced epithelial ovarian, tubal, or peritoneal cancer. Even in highly selected patients, the potential benefit of PDS remains uncertain and largely theoretical. Future studies should aim to refine patient selection, integrate molecular and imaging predictors of resectability, and reassess the role of surgery in the era of HIPEC and PARP inhibitors. Importantly, the absence of statistically significant differences should not be interpreted as proof of equivalence between PDS and NACT, given the heterogeneity of trial designs, variable statistical power, and the width of confidence intervals.

## Supplementary Materials



## Data Availability

All data and the methodological process for their calculation can be supplied under explicit request to the corresponding author and provided as an ‘.R’ file.

## Supplementary Materials

The supplementary material is available online at https://www.techscience.com/doi/10.32604/or.2026.074934/s1.

## Abbreviations

AGO-OVAR: Arbeitsgemeinschaft Gynaekologische Onkologie Studiengruppe Ovarialkarzinom
AUC: Area under the Curve
BRCA: Breast Cancer Gene (1 or 2)
CC0: Complete Cytoreductive Surgery
CHORUS: Chemotherapy OR Upfront Surgery Trial
CI: Confidence Interval
CRediT: Contributor Roles Taxonomy
DFS: Disease-Free Survival
EORTC: European Organization for Research and Treatment of Cancer
FIGO: Fédération Internationale de Gynécologie et d’Obstétrique
FUP: Follow-Up
GINECO: Groupe d’Investigateurs Nationaux pour les Études des Cancers de l’Ovaire
HIPEC: Hyperthermic Intraperitoneal Chemotherapy
HRD: Homologous Recombination Deficiency
IDS: Interval Debulking Surgery
IQR: Interquartile Range (Menzionato Implicitamente Nelle Analisi Statistiche Standard)
IRCCS: Istituto di Ricovero e Cura a Carattere Scientifico
JCOG: Japan Clinical Oncology Group
NACT: Neoadjuvant Chemotherapy
NEJM: New England Journal of Medicine
NOS: Newcastle–Ottawa Scale
OR: Odds Ratio
OS: Overall Survival
OVHIPEC: Ovarian Hyperthermic Intraperitoneal Chemotherapy Trial
PDS: Primary Debulking Surgery
PRISMA: Preferred Reporting Items for Systematic Reviews and Meta-Analyses
PROSPERO: International Prospective Register of Systematic Reviews
RCT: Randomized Controlled Trial
RR: Risk Ratio
TRUST: Trial of Radical Upfront Surgical Therapy
UK: United Kingdom

## Appendix A

**Table A1:** Newcastle–Ottawa scale.

Comparative Studies						
Registration Number	Identification Name	Country	Selection	Comparability	Exposure	Total
NCT00003636	EORTC	Europe	3	2	3	7
ISRCTN74802813	CHORUS	UK-New Zeland	3	2	3	7
UMIN000000523	JCOG0602	Japan	3	1	3	7
NCT01461850	SCORPION	Italy	4	1	2	7
NCT02828618^	TRUST	Europe	4	2	3	8

**Table A2:** Studies’ Design and Key limitation.

Trial	Design	Primary Endpoint	Assumed Effect Size (HR)	Planned/Actual Power	Surgical Quality Indicators
NCT00003636	Non-inferiority	OS	HR 1.25 (NI margin)	~80%	Low CC0 rates; heterogeneous surgical effort
ISRCTN74802813	Non-inferiority	OS	HR 1.18 (NI margin)	~60%	Low CC0 rates; limited surgical radicality
UMIN000000523	Non-inferiority	OS	HR 1.25	~70%	Higher surgical quality; underpowered
NCT01461850	Superiority	PFS	HR 0.60	~80% (optimistic)	High CC0 rates; laparoscopic selection
NCT02828618^	Superiority	OS	HR 0.75	~80%	High-volume centers; strict credentialing

Note: CC0: no residual macroscopic disease, HR: hazard ratio, OS: overall survival, PFS: progression free survival.
